# Editorial Correspondence

**Published:** 1878-09

**Authors:** 


### EDITORIAL, ETC.

### EDITORIAL CORRESPONDENCE.

ORKNEY SPRINGS, VA., AUGUST 12TH, 1878.

*My Dear Doctor.*—That was a shrewd suggestion, or remark, which came from one of my patients the other day: "You dentists," said he, don't get enough of your own breath, and too much of other people's. This condition of minus oxygen which all who handle the excavator feel to a greater or less extent, had so far run down your humble associate, as to make him feel that unless the ordinary occupations of life could be substituted for a time by something less confining, and his present breathing by something more oxygenizing than the stifling atmosphere of the city, he was in a fair way of compounding with the creditors by whose tolerance he holds a lease of life, and indeed of throwing up that lease altogether. And so, not willing to be made a martyr of, even in the blessed cause of dental science, I have made a temporary abandonment of the "means whereby we live"—*i. e.*, the office and laboratory, the chair and the lathe, and have betaken myself to the Mountains of Virginia, on the slopes of which is located the watering place whose name stands at the head of this epistle.

How rich in natural gifts is this old State, the birth place of both of us! The gold, iron, coal, minerals, plaster, are so far only conjectured of as to abundance and value; and in mineral springs as well, is she truly bestrewed. Indeed there is no country on the civilized earth where so many and so various fountains of health well up, as in this Appalachian range of mountains. Within a few yards of where I sit, are alum, chalybeate, magnesia, soda, arsenical and other springs,—half a dozen qualities of medicinal waters welling out of the mountain side, and possessing therapeutical virtues of marked value. Their effect is seen in the improving appetite, the elastic step, the clearing complexion and the sound sleep, while the pure, cool, bracing mountain air and the generous diet are truly renovating. Dyspepsia flees, the eye brightens and the spirits rise, the pulses beat fuller and stronger, and the invalid or the simply "run-down" man turns back the fast-moving shadow on the dial of time not a few degrees.

I am out of the reach of politics,, and even exchanges do not get this far. The instinct of the healer prompted me to bring a few pairs of forceps and some palliatives, for which occasional use is found, but the prime idea here is relaxation. The only hours are for meals and sleep, and the only task assiduously pursued is the swallowing of the pleasantly cool water of the "Bear-Wallow" Spring, so-called because of a tradition that in the days when the "forests primeval" covered these grounds the bears used this spring as a bathing place. The name is not suggestive of its qualities, which are tonic, diuretic and alterative, starting the kidneys and moving the liver as effectively and far more beneficially than mercurials. For "run-down" people, ænemic and chlorotic girls, and broken down women and dyspeptics, I am sure that these waters, if intelligently used, will greatly relieve.

Why cannot we, with all our knowledge, formulate compounds which will substitute these? These few grains of iron and magnesia and lime in the gallon of water seem so inadequate to the effect they certainly produce; and yet the wisdom of our chemists stands helpless to do what the tonic and alterative waters of the old and new world accomplish.

I wish our friends from the South, who a few years ago so strenuously advocated, in common with some from other places, the doctrine of the nutrition of fully formed teeth, and the dogma that the internal administration of lime-salts would avert caries, could travel around among these mountains and notice the teeth of these mountaineers who are raised on lime water. I am sure their ideas as to the prevention of decay by such means would be modified, for I have rarely seen among any rustic population, caries so widely prevalent. It is rare to see a perfect denture, even in the mouth of a young person. It is said, that some of these mineral waters contain free sulphuric acid in small quantity, but it is hardly possible that this minute quantity could injure the teeth; besides which I doubt the fact, as the tendency of this acid to form lime-salts is so great as to preclude its existence in a free form, and then too the most of these people do not drink the mineral waters, as a rule.

Two beautiful centrals which I filled four years ago, and prided myself on having done it more than ordinary well, faced me this morning, dead. The fillings were not very large, or seemingly near the pulp, and are themselves all right, but the pulps have perished. The lady is the frequent bearer of children —always has a new baby on hand. Can this condition cause, or help to cause this calamity?

But this paper has grown unconsionably, and is, I realize, prosy. The truth is, one feels little like thinking or writing here, and so I will stop.

Yours truly,

